# Navigating an Unpredictable Supply: Lived Experiences of Xylazine Exposure Among People Who Use Drugs

**DOI:** 10.21203/rs.3.rs-7199750/v1

**Published:** 2026-01-13

**Authors:** Raagini Jawa, Margaret Shang, Samia Ismail, Stephen Murray, Cristina Murray-Krezan, Yihao Zheng, Sarah Mackin, Kenny Washington, Pedro Alvarez, Jaime Dillon, Gary McMurtrie, Alexander Y. Walley, Jane M. Liebschutz

**Affiliations:** a Center for Research on Healthcare, Division of General Internal Medicine, Department of Medicine, University of Pittsburgh School of Medicine, 3609 Forbes Ave, 2^nd^ floor, Pittsburgh, PA 15213; b Harvard Medical School, 25 Shattuck St, Boston, MA 02115; c Grayken Center for Addiction, Clinical Addiction Research and Education Unit, Section of General Internal Medicine, Boston University Chobanian & Avedisian School of Medicine and Boston Medical Center, 801 Massachusetts Avenue, Second Floor, Boston, MA, 02118; d AHOPE, Boston Public Health Commission, 774 Albany St, 1st Floor, Boston, MA 02118; e Tapestry, 1985 Main Street, Suite G, Springfield, MA 01103; f Life Connection Center, 192 Appleton St, Lowell, MA 01852; g Geisinger Commonwealth School of Medicine, 525 Pine St, Scranton, PA 18509

**Keywords:** harm reduction, xylazine, adulterants, wounds, people who use drugs

## Abstract

**Background:**

As xylazine-adulterated opioids become more prevalent in the U.S., people who use drugs (PWUD) face growing risks from sedation, withdrawal, and wounds. This study explores PWUD perceptions on recognizing xylazine exposure including its physical effects and clinical harms and how these factors shape their drug use practices.

**Methods:**

In August 2023, we surveyed adult PWUD clients reporting at least one past-year drug use-related wound across three Massachusetts syringe service programs with high xylazine prevalence. We compared demographics, drug use factors, physical effects, and clinical symptoms between those with and without self-reported past-90-day xylazine exposure and conducted content analysis of open-ended responses.

**Results:**

Of the 171 respondents, 80% (n=136) reported past-90-day xylazine exposure. The majority of respondents were male, white, non-Hispanic, and aged 36–45 years, with no significant differences by xylazine exposure. Xylazine-exposed participants commonly reported sedation (77%), loss of consciousness (52%), and wounds (91%). Most participants were afraid and frustrated, seeing xylazine as an unwanted contaminant but were often unable to avoid it due to financial constraints, withdrawal symptoms, and limited alternative options.

**Conclusion:**

Syringe service program clients in Massachusetts commonly reported xylazine-adulterated fentanyl exposure, recognized through heavy sedation and skin wounds. Their experiences highlight the urgent need for real-time xylazine detection, safer supply, overdose and sedation risk mitigation, and improved wound prevention and care.

## Background:

Xylazine is a veterinary sedative increasingly adulterating the US unregulated opioid supply since 2019(1) with a particularly high concentration in the northeastern US.(2–4) Xylazine adulteration was first documented in the early 2000s in Puerto Rico, primarily in the unregulated heroin supply,(5, 6) but has since spread widely along with the replacement of heroin by fentanyl. In 2023, xylazine was detected in 38–49% of lab tested fentanyl samples in Massachusetts.(7) As its prevalence grows, people who use drugs (PWUD) are both intentionally and unintentionally exposed, raising concerns about higher morbidity and mortality due to emerging clinical harms,(6, 8, 9) including xylazine-associated wounds.(10, 11) The scope of xylazine-related health effects remains largely anecdotal, based on reports from frontline clinicians in regions with high rates of adulteration.(12)

Prior to the availability of community-based drug checking(13) PWUD often relied on physical cues to detect fentanyl adulteration in the unregulated opioid supply and to differentiate it from heroin.(14) Fundamental harm reduction overdose prevention strategies that PWUD used included test dosing (i.e., using a smaller amount first to test potency), taking turns with peers, and more recently utilizing fentanyl test strips when they are available.(15, 16) However, it remains unclear whether distinct physical cues or clinical harms can reliably signal xylazine exposure, highlighting a critical gap in harm reduction knowledge as xylazine continues to spread.

Given the continued volatility of the unregulated drug market and shifting potency levels, more direct, first-hand accounts from PWUD about their experiences with xylazine are needed to guide xylazine related-harm reduction efforts. The primary objective of this study was to explore the experiences of individuals with self-reported xylazine exposure—examining how they recognize or suspect exposure, the clinical symptoms and physical effects they experience, and how these factors influence their drug use practices.

## Methods:

### Setting and recruitment

We conducted a cross-sectional survey of a convenience sample of PWUD from three Massachusetts community-based syringe service programs (SSPs) in Lowell, Boston, and western Massachusetts. These medium to large SSPs serve racially and ethnically diverse PWUD populations. PWUD were eligible if they were adults with one or more self-reported past-year drug use-related wounds. We did not exclude PWUD on the basis of route of administration.

Over a four-week period in August 2023, we recruited eligible participants through flyers posted in each SSP or directly via word-of-mouth. We aimed to recruit a convenience sample of at least 50 participants per SSP, a target which accounted for each site’s client volume and feasibility of onsite data collection while minimizing operation disruptions. Research staff directed potential participants to either a secure onsite computer to fill out the survey via Qualtrics or distributed a paper version of the survey. A research assistant and SSP staff were present to troubleshoot technical issues and support data collection including transcribing open-ended responses. Survey participants and SSP sites were compensated $25 gift cards and $500 stipend, respectively.

### Survey instrument

We developed a 33-item survey instrument composed of multiple-choice, free response, and visual identification questions (See Appendix). Frontline SSP staff and substance use care providers with xylazine content expertise reviewed the instrument for face validity. The survey was translated into Spanish via an independent professional certified translation service and reviewed by bilingual study team members and SSP staff. This manuscript is focused on analyzing participants’ physical experiences with xylazine and clinical outcomes associated with xylazine exposure. We previously published an analysis of drug use and wound care practices and treatment experiences using a subset of this data.(17)

The survey recorded participant characteristics including gender, race/ethnicity, housing status, and treatment history with medications for opioid use disorder. Using the modified Addiction Severity Index,(18) we also assessed substances used, substance use frequency with active use defined as using a substance a few times a week or more, and route(s) of use (injection, intranasal, oral, per-rectal, or smoking) including their primary route. For those that primarily injected, participants reported areas they typically injected via a hotspot question (image with selectable areas). Participants who reported exposure to xylazine were labeled “xylazine exposed”, and all others labeled “xylazine non-exposed” including those unsure whether they had exposure. Participants then gave a rationale for what made them report exposure or non-exposure (i.e., xylazine exposure/non-exposure confirmed via urine testing, etc.) and whether they had experienced new clinical symptoms or post-use physical effects when using substances (e.g., sedation, wounds, nasal ulcers, etc.). Human and veterinary literature and experiences of frontline SSP workers informed options for these questions as well as questions on anticipated behavioral responses to xylazine adulteration.(19, 20) Participants could further elaborate on via free text.

Although no standard clinical definition of xylazine-associated wounds exists, descriptions in the literature and clinical experience indicate a spectrum of severity, ranging from red-purple blisters to large ulcers with or without eschar.(21) Based on these descriptions, the study team developed three illustrations representing this spectrum and depicting a range of skin tones. Then they presented these illustrations to participants, who visually identified any similar lesions they had experienced in the past 90 days. The team also asked participants whether they had experienced wounds at non-injection site and instructed them to indicate the anatomic locations of all drug-use-related wounds using hot-spot mapping on anterior and posterior body diagrams. The study team characterized participants based on the overlap between wound and injection sites as follows: those in the *lnjection Only* group had wounds exclusively at injection sites; those in the *Non-lnjection Only* group had wounds that did not overlap with any injection sites; and those in the *lnjection and Non-injection Site* group had at least one wound at an injection site and one at a non-injection site.

Throughout the survey, participants could elaborate on their experiences with xylazine and drug use-related wounds via optional free-text responses. Participants provided these open-ended responses either in writing or by dictating them to SSP staff or a research assistant, who transcribed them verbatim during survey administration. We used the past 90 days as the time period for all variables. This study was approved by the University of Pittsburgh Institutional Review Board.

### Data Analysis

We used descriptive statistics to describe categorical variables through frequencies and proportions. Using Fisher’s exact and chi-squared tests, we evaluated between-group differences for “xylazine exposed” versus “xylazine non-exposed” participants. We calculated frequencies of participant reasons for suspected xylazine exposure and anticipated responses to xylazine adulteration. For participants with self-reported xylazine exposure, we used a Venn diagram to illustrate the frequency and overlap of xylazine-related wound types reported in the past 90 days to illustrate the overlap between multiple wound types, highlighting patterns of co-occurrence that may not be apparent with other visualization methods. For those who primarily reported using substances via injection, we generated heatmaps to visually compare the prevalence of 90-day wounds at injection versus non-injection sites. because they clearly display spatial patterns and intensity of wound prevalence, allowing for easy comparison across anatomical locations. All quantitative analysis was performed in R Version 4.3.1.(22) Finally, two study team members (RJ, MS) inductively coded free-text responses to capture the nuanced lived experiences of participants using Nvivo (Lumivero, version 14, 2023). The team reviewed the adjudicated codes to derive representative quotations to supplement quantitative results through content analysis.

## Results

A total of 175 PWUD participated in the survey including 75 from the Boston site and 50 each from the Lowell and western Massachusetts sites of which 171 surveys were >25% complete and comprise the study sample. 47% (n=80) of participants provided an open-ended response. The majority of participants were male (63%), 36 to 45 years of age (41.5%), white (62.6%), non-Hispanic (67.3%), and unhoused (72.5%). The most frequently used substances included fentanyl/heroin (93%) and cocaine (73.7%), with almost 80% of participants reporting xylazine exposure in the past 90 days. There were no significant differences in participant characteristics between the xylazine exposed and xylazine non-exposed groups (Table 1). Among those who had self-reported xylazine exposure, 47.8% experienced injection site wounds, 36% non-injection site wounds, and 8.1% with both.

### Reasons for suspecting xylazine exposure

**Figure 1:**
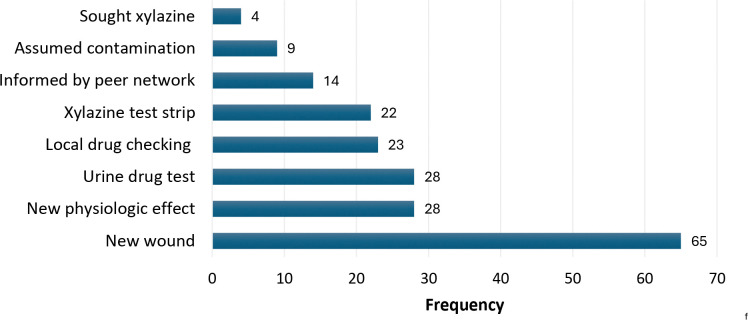
Reasons participants suspected xylazine exposure (N=136)^a^ ^a^Categories are not mutually exclusive as respondents could select more than one option;35 participants did not report xylazine exposure.

Among the 136 participants reporting xylazine exposure, the most common reason for suspecting xylazine exposure was experiencing a new wound (Figure 1). One participant described how these wounds were distinct from typical drug use-related skin and soft tissue infections (SSTIs): “*The way the wounds came out; it was a different kind of abscess, and they were coming out everywhere. It was coming out like a pimple at first. And they get bigger and bigger and they almost like eat your flesh.”* A fifth of participants reported experiencing a new physical effect as a reason for suspecting xylazine exposure, expanding on these effects in the free-text responses as feeling a different taste in their mouth, a burning sensation when injecting, severe sedation, or loss of consciousness after use. One participant said, “*I get more sleep standing up now than lying down… I have new wounds and it knocks you the fuck out and it is a very unpleasant high. You get a little bit of a rush and the next thing you know I am standing sleeping for an hour.”* A similar proportion of participants reported verifying suspected xylazine exposure from varied types of drug testing including urine toxicology, local drug checking services, or xylazine test strips. A minority of respondents assumed xylazine exposure given the ubiquity of xylazine contamination in the unregulated opioid supply with one elaborating, “*It’s almost everywhere now, it just has to be and then you don’t know if you get it*.”

At the time of the study, only four participants reported xylazine exposure from intentionally seeking xylazine-adulterated opioids. One participant said, “*I like it. I will do whatever it takes to numb me. That’s what I’m after… I first heard about it in 2008, and they were selling it [in Puerto Rico], and I went over there to buy it. People were using it back then to cut heroin there. [They] mostly were getting it from veterinar*ians.”

Among the 35 participants without reported xylazine exposure, 28 were unsure of being exposed while seven were certain of non-exposure. Reasons ranged from no prior knowledge of xylazine, assumption their heroin did not contain xylazine, negative urine drug test for xylazine, or not experiencing any physical effects or wounds related to xylazine.

### Anticipated responses to xylazine adulteration

**Figure 2:**
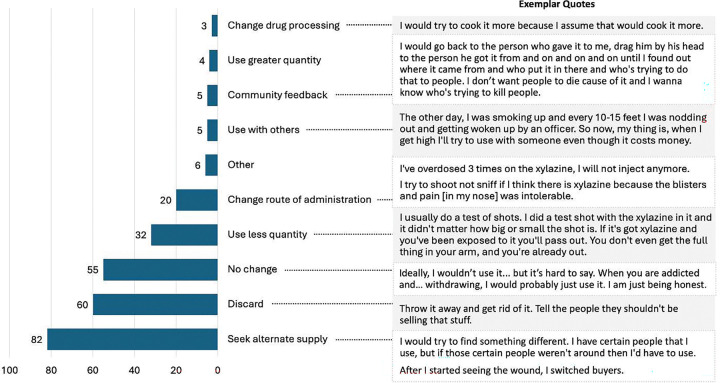
Anticipated responses to confirmed xylazine adulteration with exemplar quotes (N=171)^a^ ^a^Categories are not mutually exclusive; respondents could select more than one option.

When asked how they would react if their drugs tested positive for xylazine, all participants—regardless of whether they reported known exposure to xylazine—described a range of anticipated responses, with the majority indicating they would discard the drug and/or seek an alternative supply (Figure 2). Others reported making no changes due to the lack of alternative options in the drug supply, financial limitations to obtain a new supply, or immediate needs to treat active withdrawal. Others expressed modifying their drug use practices to minimize negative effects or risks associated with xylazine through employing harm reduction strategies such as using a smaller amount, using with others, or changing the route of administration. Several participants provided detailed explanations of their anticipated responses, as illustrated in Figure 2.

### Clinical symptoms and post-use physical effects

The most frequently experienced physical effect post-use among those with self-reported xylazine exposure was sedation (77.2%) and loss of consciousness (51.5%, Table 2). Individuals who were xylazine exposed experienced higher rates of new clinical symptoms compared to those non-exposed: 68% versus 60% reported dry mouth, 49% versus 26% reported a productive cough, and 43% versus 31% reported nasal ulcerations, respectively.

In terms of primary route of administration, those primarily using drugs intranasally reported nasal ulceration or sinus pain (65%) as the most common new clinical symptoms. Individuals who primarily smoked reported dry mouth (88%) followed by productive cough (75%), while individuals who primarily injected reported, dry mouth (65%) followed by wounds (42%). Loss of consciousness was more common among participants whose primary route of drug use was smoking (50%) or intranasal (59%), compared to those who primarily injected (44%).

**Figure 3:**
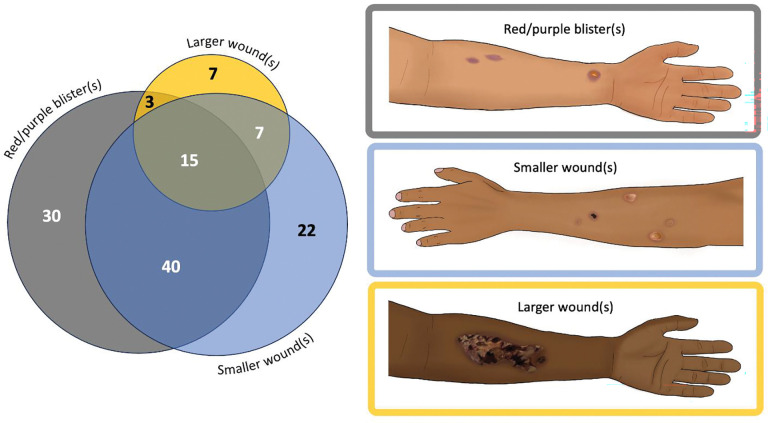
Past 90-day xylazine wound type experienced by PWUD with self-reported xylazine exposure represented by Venn diagram (N=124)

Among participants with self-reported xylazine exposure, 91% (n=124) recalled experiencing a xylazine wound(s) within the past 90 days. The majority reported early-stage xylazine wounds, typically presenting as red/purple blisters and/or smaller wounds (Figure 3). Only a minority reported larger, more complex wounds.

**Figure 4.**
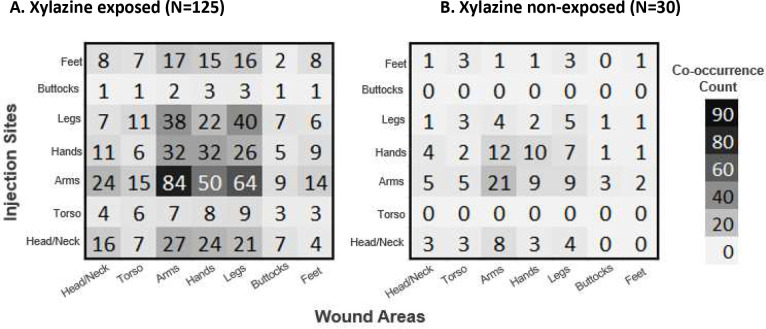
Co-occurrence matrix as a heatmap between wound location and injection site among people primarily using via injection (N=155)^a^ ^a^Categories are not mutually exclusive with participants able to select all the areas in which they most frequently inject as well as all the areas in which they experienced drug use-related wounds in the past 90 days. Co-occurrence count reflects the frequency in which the reported wound anatomic location and injection site are the same for one participant.

Among participants who primarily injected drugs in the past 90 days (n=155), those who reported xylazine exposure most commonly injected in their hands, arms, and legs, with high frequencies of wound co-occurrence in these areas (Figure 4). Participants without known xylazine exposure showed similar injection and wound patterns. Although wound locations generally aligned with common injection sites across all participants, those with xylazine exposure had a higher proportion of leg wounds despite injection in their arms, compared to those without exposure (51% vs 30%, respectively).

### Fear, frustration, and the need for control in an unregulated drug supply

Most participants widely expressed fear and frustration about the unpredictable and toxic drug supply, especially with xylazine. Many described xylazine as unwanted and a dangerous adulterant, calling it “*straight poison*,” and voiced concerns about its potential unknown long-term effects: “*If it’s already doing this to our skin, I can’t imagine what it’s doing inside*.” This fear often extended beyond xylazine to the broader drug supply, highlighting the psychological toll of navigating an unregulated and unpredictable drug supply. One expressed, “*I’m scared of drugs right now, I’m losing my mind, forgetting things, not able to control the effects. Trying to figure out if the xylazine is in the coke or in the heroin, so I can figure out which drugs are safe to use*.” For some, their Frustration stemmed from the lack of safer alternatives and deceptive seller practices, which left participants feeling trapped in a harmful cycle: “*It’s hard to buy from a different seller because all of the sellers [are] from the same source... and some sellers will use food coloring so you don’t see the difference*.” Despite these fears, some participants continued use out of necessity, with one saying, “*I’m scared to use but need to. Don’t wanna end up with really bad wounds*.” Several expressed a desire to regain control or stop using altogether, underscoring the urgent need for a safer, regulated supply, with one saying, *“l want to stop using drugs because I think they’re going to kill me in the end. Tranquilizer? I’m not a horse.”*

## Discussion

Our cross-sectional embedded design mixed-method study is one of the first to investigate the experiences of a convenience sample of PWUD with past-year drug use-related wounds regarding the changing drug supply in an area with high prevalence of xylazine adulteration. We found that the majority of our sample reported exposure to xylazine, primarily through experiencing new clinical symptoms and physical effects, which have been previously described in the literature.(20) We found that these clinical manifestations such as heavy sedation and wounds of varying severity acted as indicators of the changing drug supply more so than drug testing, reflecting similar experiences of PWUD during the emergence of fentanyl.(14, 15) Early data from other studies also shows that PWUD rely on subjective health effects to determine whether they have been exposed to xylazine.(23) Relying on post-use effects to detect changes in drug supply places PWUD at unnecessary risk for a range of serious outcomes beyond overdose, including injury, assault, complex wounds, secondary infections, and, in severe cases, amputation.(20)

Our findings indicate that the development of new wounds was frequently a sentinel sign of xylazine exposure.(24) Locations of these wounds varied but were primarily found on extremities. Moreover, consistent with prior literature, individuals reporting xylazine exposure experienced wounds even with non-injection routes of drug administration and at non-injection sites across the body, suggesting potential systemic effects of xylazine beyond localized trauma.(25) Additional research is warranted to evaluate the pathophysiology behind developing non-injection site wounds. Most individuals with xylazine-related wounds, as classified by visual identification, had early-stage red/purple blisters or coin-sized lesions,(26) highlighting a critical window for timely intervention before wounds progress to more severe complex ulcers, which has been more frequently described and emphasized in existing literature.(27–29) Furthermore, some participants who altered their route of use experienced additional harms; for example, those who switched to intranasal use developed nasal ulcers and sinusitis, aligning with prior reports.(20, 30) These findings point to the urgent need for research into the mechanisms of xylazine-related tissue damage beyond injection-related wounds. Moreover, these findings imply that traditional harm reduction strategies such as route transitioning (i.e., switching from primarily injecting to smoking, etc.) may not be applicable. While our sample size was not large enough to determine statistically significant associations between primary routes of use and symptomatology (e.g., nasal ulcers with primary intranasal use), our results highlight the need for further research to explore these potential correlations to inform best practices from a harm reduction perspective.

Despite the need to determine optimal approaches to reduce harm to xylazine exposure, we found that PWUD actively employed strategies to navigate the unpredictable unregulated drug market and address associated harms. Contrary to portrayals of PWUD as passive consumers incapable or unwilling to reduce health risks, our findings illuminate PWUD’s agency in proactively making adaptations to decrease drug-associated harms. Proposed modifications to enhance safety included having other individuals monitor them post-use, changing dealers to find more consistent or less adulterated products, and shifting routes of administration—from injection to smoking or intranasal use—to minimize sedation, prevent wounds, and better manage drug effects.

Even with access to xylazine test strips and drug checking resources at the study sites, some participants remained uncertain about their xylazine exposure. This highlights both the underuse of and growing need for accessible drug-checking technologies.(31) Furthermore, while PWUD have a growing interest in using xylazine test strips,(32, 33) their effectiveness is limited by cost, legal restrictions, and performance variability, necessitating their use alongside more advanced tools.(34, 35) Point-of-care drug checking using spectroscopy offers accurate, real-time substance identification and direct engagement with PWUD, reducing dependence on harmful physical cues and supporting more informed, safer use.(23)

Moreover, even with drug checking, PWUD expressed frustration over the ubiquity of xylazine in their supply and the absence of basic protection from the increasingly toxic and unregulated drug market. Many expressed a strong desire for safer alternatives and greater control over their consumption or stopping using drugs entirely, highlighting the lack of options for non-adulterated drugs due to prevailing market forces.(23, 32, 36, 37) This lack of agency is not an indication of personal failing but as a symptom of criminalization of drugs and structural neglect. Addressing the current crisis requires reimagining drug policy to prioritize the human rights and safety of PWUD.(38) Participants emphasized the need for evidence-based solutions to reduce exposure to toxic adulterants, lower overdose risk, improve health and social outcomes, and prioritize individual choice and health over punitive approaches.(39, 40) As new adulterants continue to emerge, there is a pressing need to evaluate these grassroots practices in partnership with harm reduction organizations, while also expanding their capacity to deliver comprehensive drug user health services. This includes safe consumption spaces,(41) distribution of safer smoking and snorting supplies,(42) drug checking technologies, wound care resources, and timely, accurate information about emerging contaminants and risk mitigation practices. Supporting PWUD’s autonomy is essential to building responsive, effective public health efforts.

While this exploratory study offers important insights into the lived experiences of PWUD amid an increasingly toxic and unpredictable drug supply, it is limited by its reliance on self-reported xylazine exposure without toxicological confirmation, which may have led to some misclassification. However, previous research has found self-report of xylazine exposure to be somewhat reliable among PWUD.(23, 43) Second, the accuracy of reports on wound onset, location, and patterns of use may have been affected by recall bias and confounded by the high prevalence of polysubstance exposure. Third, while nearly half of participants offered detailed, open-ended responses that enriched the data, the findings remain subject to individual memory and interpretation. Finally, as a cross-sectional study, this research does not capture the long-term effects of xylazine exposure, wound progression, or changes in drug use practices over time—factors that are critical to shaping effective clinical and harm reduction responses. Despite these limitations, the study contributes valuable perspectives to a growing body of research and underscores the importance of centering community expertise in addressing emerging drug trends.

## Conclusion:

This study sheds light on the lived experiences of PWUD who suspect xylazine exposure, offering valuable insights into how they identify exposure, interpret its physical effects and clinical symptoms, and modify their drug use practices in response. These first-hand accounts highlight the urgency of expanding access to drug checking technologies, safer supply options, and tailored harm reduction strategies. As traditional surveillance and clinical data often lag behind emerging drug trends, centering the voices of PWUD is essential for crafting timely, responsive interventions that address the evolving risks of the current drug landscape.

## Supplementary Material

Supplementary Files

This is a list of supplementary files associated with this preprint. Click to download.

• JawaHRJSupplement.docx

## Figures and Tables

**Table 1:** Characteristics of PWUD with and without past 90-day self-reported xylazine exposure

	All N=171	Xylazine exposed N=136 (79.5%)	Xylazine non-exposed N=35 (20.5%)	P-value
**Gender**				
Female	61 (35.7%)	51 (37.5%)	10 (28.5%)	0.21^a^
Male	108 (63.2%)	84 (61.8%)	24 (68.6%)	
Transgender/Non-binary/Other	2 (1.2%)	1 (0.7%)	1 (2.9%)	
**Age**				
18–25	6 (3.5%)	4 (2.9%)	2 (5.7%)	0.29^a^
26–35	46 (26.9%)	40 (29.4%)	6 (17.1%)	
36–45	71 (41.5%)	57 (41.9%)	14 (40%)	
46–55	34 (19.9%)	26 (19.1%)	8 (22.9%)	
>55	14 (8.2%)	9 (6.6%)	5 (14.3%)	
**Race**				
Black	18 (10.5%)	15 (11.0%)	3 (8.6%)	0.18^a^
American Indian, Alaskan Native, Native Hawaiian, or Asian	3 (1.8%)	3 (2.2%)	0 (0.0%)	
Multiracial	10 (5.8%)	9 (6.6%)	1 (2.9%)	
White	107 (62.6%)	88 (64.7%)	19 (54.3%)	
Prefer not to say	33 (19.3%)	21 (15.4%)	12 (34.3%)	
**Hispanic**	56 (32.7%)	40 (29.4%)	16 (45.7%)	0.10^b^
**Unhoused** ^c^	124 (72.5%)	100 (73.5%)	24 (68.6%)	0.71^b^
**Active substance used** ^d^				
Fentanyl/Heroin	159 (93.0%)	128 (94.1%)	31 (88.6%)	0.27^a^
Cocaine	126 (73.7%)	99 (72.8%)	27 (77.1%)	0.76^b^
Methamphetamine	30 (17.5%)	26 (19.1%)	4 (11.4%)	0.33^a^
Benzodiazepines	42 (24.6%)	33 (24.3%)	9 (25.7%)	1.00^b^
Non-prescription opioids	18 (10.5%)	14 (10.3%)	4 (11.4%)	0.77^a^
**MOUD treatment**	89 (52.0%)	71 (52.2%)	18 (51.4%)	1.00^b^
**Past 90-day wounds**				
Injection site only	79(46.2%)	65 (47.8%)	14 (40.0%)	0.68^b^
Non-injection site only	62 (36.3%)	49 (36.0%)	13 (37.1%)	
Injection and non-injection site	14 (8.2%)	11 (8.1%)	3 (8.6%)	
Never injected	16 (9.4%)	11 (8.1%)	5 (14.3%)	

Abbreviations: MOUD, medication for opioid use disorder; PWUD, people who use drugs

a P-value calculated from the Fisher’s Exact test.

b P-value calculated from the Chi square test.

c Unhoused (e.g., currently lives in a shelter or have no steady place to sleep at night)

d Active substance use is defined as using at least a few times a week based on the Addiction Severity Index.

**Table 2:** Post-use physical effects and new clinical symptoms in the past 90 days among PWUD by self-reported xylazine exposure and primary route of use (N=171)^a^

	All N=171	Xylazine exposure		Primary route of drug use		
		Exposed N=136 n (%)	Non-exposed N=35 n (%)	Injection N=138 n (%)	Smoking N=16 n (%)	Intranasal N=17 n (%)
**Post-use physical effect**						
**Sedation**	126 (73.7%)	105 (77.2%)	21 (60.0%)	100 (72.5%)	14 (87.5%)	12 (70.6%)
**Loss of consciousness**	78 (45.6%)	70 (51.5%)	8 (22.9%)	60 (43.5%)	8 (50.0%)	10 (58.8%)
**New clinical symptom**						
**Nasal ulcer/sinus pain**	70 (40.9%)	59 (43.4%)	11 (31.4%)	52 (37.7%)	7 (43.8%)	11 (64.7%)
**Productive cough**	76 (44.4%)	67 (49.3%)	9 (25.7%)	57 (41.3%)	12 (75.0%)	7 (41.2%)
**Dry mouth**	114 (66.7%)	93 (68.4%)	21 (60.0%)	90 (65.2%)	14 (87.5%)	10 (58.8%)
**Anemia** ^b^	34 (19.9%)	28 (20.6%)	6 (17.1%)	30 (21.7%)	2 (12.5%)	2 (11.8%)
**Wounds**	71 (41.5%)	56 (41.2%)	15 (42.9%)	58 (42.0%)	7 (43.8%)	6 (35.5%)
**Anal ulcers**	7 (4.1%)	7 (5.1%)	0 (0.0%)	6 (4.3%)	0 (0.0%)	1 (5.9%)
**Dysglycemia** ^c^	36 (21.1%)	29 (21.3%)	7 (20.0%)	28 (20.3%)	5 (31.2%)	3 (17.6%)
**Other** ^d^	20 (11.7%)	16 (11.8%)	4 (11.4%)	13 (9.4%)	3 (18.8%)	4 (23.5%)
**No symptoms**	10 (5.8%)	6 (4.4%)	4 (11.4%)	7 (5.1%)	3 (0.0%)	3 (17.6%)

a Categories are not mutually exclusive; respondents could select more than one option. Frequencies may sum to more than the total sample size.

b Anemia was defined as low red blood cell counts.

c Dysglycemia was defined as high or low blood sugar.

d Other: Participants reported a range of other symptoms including respiratory issues (chronic runny nose, difficulty breathing), cardiovascular and systemic effects (heart pounding, high blood pressure, fatigue, depression), skin complications (persistent skin irritation, abscesses, hives), gastrointestinal problems (stomach pain, diarrhea), and headaches.

## Data Availability

The data that support the findings of this study may be available from the authors upon reasonable request and with permission of University of Pittsburgh.

## Abbreviations:

PWUD: People who use drugs
SSTI: skin and soft tissue infections
SSP: syringe service programs
MOUD: medications for opioid use disorder
